# Subclinical infection combined with surgery induced cognitive dysfunction: a novel adult mouse model for perioperative neurocognitive disorder

**DOI:** 10.3389/fnagi.2025.1691681

**Published:** 2026-01-12

**Authors:** Chenchen Xia, Xiao Zhang, Wanbing Dai, Yizhe Zhang, Ye Liu, Xiangyang Cheng, Yeke Zhu, Lili Huang, Minghao Tang, Yongxing Yao, Xuwu Xiang, Weifeng Yu, Diansan Su

**Affiliations:** 1 Department of Anesthesiology, School of Medicine, Renji Hospital, Shanghai Jiaotong University, Shanghai, China; 2 Key Laboratory of Anesthesiology (Shanghai Jiaotong University), Ministry of Education, Shanghai, China; 3 Second School of Clinical Medicine, Binzhou Medical University, Yantai, Shandong, China; 4 Department of Anesthesiology, The First Affiliated Hospital, Zhejiang University School of Medicine, Hangzhou, China; 5 The First People’s Hospital of Jiashan, Jiashan Hospital Affiliated with Jiaxing University, Jiaxing, Zhejiang, China

**Keywords:** perioperative neurocognitive disorder, adult mice, subclinical infection, cognition, primed immune system

## Abstract

**Introduction:**

Perioperative neurocognitive disorder (PND) describes a range of cognitive impairments associated with surgery and anaesthesia, often driven by neuroinflammation. This study explored a novel adult mouse model, in which preoperative subclinical infection, induced by low-dose lipopolysaccharide (LPS) in combination with surgery, led to cognitive dysfunction in adult mice.

**Methods:**

Adult male C57BL/6J mice were treated with 0.75 mg/kg LPS two hours before undergoing tibial fracture fixation or appendicectomy. Spontaneous activity and anxiety-like behaviours were tested by open field test. Cognitive outcomes were evaluated using the novel object recognition test and morris water maze. Inflammatory markers and synaptic proteins in the hippocampus were analysed through ELISA, RT-qPCR, and Western blot, while proteomics provided deeper insights into molecular changes.

**Results:**

We found that preoperative LPS sensitised the immune system, leading to heightened neuroinflammation and microglial activation after surgery. This was accompanied by memory and learning impairments. Key synaptic proteins, including PSD-95, GAP-43, SYN and mature BDNF, were significantly reduced, indicating disrupted synaptic function. Proteomics revealed changes in pathways related to immune responses, synaptic organisation, and energy metabolism, providing a potential molecular basis for these cognitive deficits.

**Discussion:**

This study provided a practical adult mouse model for PND, demonstrating that low-dose LPS followed by surgery induced an inflammatory response, leading to postoperative impairments in learning and memory.

## 1 Introduction

Perioperative neurocognitive disorder (PND) encompasses a range of cognitive impairments identified during the perioperative period (Barreto Chang and Maze, 2022; Barreto Chang et al., 2023). Prior to its renaming in 2018, PND was referred to as postoperative cognitive dysfunction (POCD), a term that primarily described prolonged postoperative cognitive changes rather than the acute and temporary manifestations associated with POD (Evered et al., 2018). Cognitive impairments are the hallmark of PND, including deficits in memory, attention, and social interaction. Key risk factors for PND include surgical trauma, anesthesia, and aging, with aging recognized as the most critical contributing factor. The combined impact of surgery and aging, referred to as “double stress,” contributes to cognitive decline. Although aging is considered an independent risk factor for PND, studies have shown that it can occur at any age. A study by Monk et al. reported that at hospital discharge, PND was observed in 36.6% of young patients (18–39 years), 30.4% of middle-aged patients (40–59 years), and 41.4% of elderly patients (60 years and older) (Monk et al., 2008). Current animal models for studying PND, such as peripheral orthopedic or appendectomy surgeries, were typically conducted on aged animals. The absence of an established animal model for PND in adults presents a challenge for investigating its underlying mechanisms and developing effective prevention strategies for young individuals. It is important to investigate the conditions under which PND occurs in adults and to establish a PND model in adult mice.

Studies have shown that adult individuals who develop PND exhibit higher levels of inflammatory cytokines (Barreto Chang and Maze, 2022), whereas aging itself is accompanied by chronic neuroinflammation (Andronie-Cioara et al., 2023). Systemic infections increase the levels of pro-inflammatory cytokines in the brain (Cibelli et al., 2010; Rosczyk et al., 2008), which may further impair memory consolidation, as demonstrated in animal studies (Barrientos et al., 2009; Barrientos et al., 2002). Among the pro-inflammatory mediators, interleukin IL-1β is considered a key factor driving the inflammatory response in the brain (Cibelli et al., 2010; Barrientos et al., 2009; Barrientos et al., 2002) Additionally, tumor necrosis factor TNF-α, interferon IFN-γ, and interleukin IL-6 also play significant roles in the initiation and progression of the inflammatory process (Monk et al., 2008; McAfoose and Baune, 2009; Wan et al., 2007). These findings indicate that modulating inflammatory responses may be a promising approach to alleviating PND. Elevated preoperative inflammation or a primed immune system plays a critical role in the onset of PND in adults.

Lipopolysaccharide (LPS), a major component of the outer membrane of Gram-negative bacteria, is widely recognized for its ability to trigger immune response (Banoub et al., 2010). Studies have demonstrated that priming the immune system with a subclinical dose of LPS can significantly enhance the pro-inflammatory response to subsequent LPS challenges (O’Dea et al., 2009; Finsterer, 2019). Based on this, we hypothesized that subclinical infection induced by LPS exposure may sensitize the immune system and subsequent peripheral surgery could lead to cognitive decline.

To test these hypotheses, we evaluated the degree to which presurgical LPS infection could influence the levels of inflammatory factors in hippocampus and plasma. After determining an appropriate subclinical dose of LPS as the presurgical dosage, surgery is performed, and cognitive function is subsequently measured. Spontaneous activity and anxiety like behaviors were tested by open field test (OFT). Hippocampal cognition function impairment was assessed using novel object recognition (NOR) and Morris water maze (MWM). Inflammatory factors in plasma and hippocampus were measured using ELISA and RT-qPCR. Several kinds of synaptic protein in hippocampus were measured by RT-qPCR and western blot.

## 2 Materials and methods

### 2.1 Animals

Eight-week-old male C57BL/6J mice were supplied by the Animal Research Centre of Shanghai Jiao Tong University School of Medicine. The animals were housed in standard cages under controlled laboratory conditions (temperature of 22 °C ± 2 °C, 12-h light/12-h dark cycle) with free access to standard rodent pellets and water. All mice were allowed to acclimatize to their new environment for 7 days before commencing the experiments. The mice were randomly allocated into groups and housed in separate cages to prevent interactions that might influence treatment-dependent differences.

### 2.2 Mouse surgery and treatment

All surgeries were conducted under isoflurane anesthesia using the methods described in our previous studies (Batista et al., 2019; Dunn, 2006). Specifically, anesthesia was induced and maintained with 2% isoflurane in 21% oxygen in a transparent acrylic chamber. For the LPS and surgery group, LPS was administered via intraperitoneal injection into the mice 2 hours before surgery. During the procedure, the temperature was maintained between 36 °C and 37 °C with the aid of warming pads. A single dose of butorphanol (0.4 mg/kg, s.c.) was administered for analgesia after anesthetic induction and before the skin incision. Aseptic techniques were employed throughout the procedure, and mice recovered from anesthesia within 20 min. No hypoxia (SpO_2_ < 90%) was observed during the procedure.

#### 2.2.1 Tibial fracture fixation surgery (TF)

The aseptic open tibial fracture with intramedullary fixation was performed under isoflurane anesthesia (including 2% isoflurane in 21% FiO₂), as previously described (Dunn, 2006). Briefly, the right hind paw was shaved and disinfected. A median paw incision was then made, followed by the insertion of a 0.38 mm pin into the tibial intramedullary canal. The periosteum was then stripped, and an osteotomy was performed. Following the fracture surgery, the wound was irrigated, and the skin was sutured with 4-0 Vicryl sutures. Thereafter, the animals were allowed to recover spontaneously from the anesthesia. Learning and memory were assessed using the Morris Water Maze (MWM) 4 days after surgery.

#### 2.2.2 Appendectomy (APP)

Mice underwent a standard surgical procedure for appendicectomy, in accordance with the procedures reported in a previous study (Zhang et al., 2016). First, a small incision of approximately 1 cm was made in the middle abdominal quadrant, followed by immobilization and isolation of the end of the caecum and appendix. Two ligatures were placed proximal to the border of the appendix and the caecum, and division of the appendix was performed between the two ligatures. To ensure the smooth function of the intestine, at least two thirds of the original appendix length was retained. The caecal stump and abdominal cavity were flushed with saline, and the intestine was returned to its position. Finally, the two layer abdominal wall was closed.

### 2.3 Behavioral tests

All behavioral tests were conducted during the light period between 9 a.m. and 6 p.m., and the apparatus was carefully cleaned by wiping with 70% ethanol after each trial to remove olfactory cues. All mice were familiarized with the researchers and habituated to the training room prior to the experiment. The animals to be tested were moved into the behavioral testing room 30 min before the start of the trial to minimize olfactory cues. The bedding of the animals’ home cages was not changed during the period of the behavioral tests. To minimize the variation introduced by experimenter handling, the mice were handled by the same experimenter throughout all behavioral experiments.

#### 2.3.1 Open field test (OFT)

Spontaneous activity and anxiety like behaviors in the open field were assessed using an opaque plastic cube box (40 × 40 × 40 cm) under a camera; the center region was defined as a 15 cm × 15 cm area. To reduce anxiety in the animals, the light intensity in the center of the box was set to 100 ± 5 lux. Each mouse was first placed in the center of the box and given 15 min to move freely. Mean speed and time spent in the center area were recorded and analyzed.

#### 2.3.2 Novel object recognition (NOR)

The NOR test was conducted to assess short term recognition memory. Each trial consisted of a familiarization session and a test session. In the familiarization session, each mouse was placed into a testing box (40 × 40 × 40 cm); to reduce anxiety in the animals, the light intensity in the center of the box was set to 100 ± 5 lux. The mouse was allowed to explore freely for 10 min in the box, which contained two identical objects (5 × 5 × 5 cm) at symmetrically diagonal positions. One hour later, one familiar object in the box was replaced with a novel object of the same size but different brightness, shape, and texture. In the test session, the mouse was placed back into the box and given 10 min to explore the two objects. Animals that remembered the familiar object spent more time exploring the novel object. The time spent sniffing and exploring the novel object was recorded as the period when the tip of the snout was near the object (distance between the nose and the object was less than 2 cm). The discrimination index was defined as the ratio of exploration duration around the novel object to the duration around both objects in the test session.

#### 2.3.3 Morris water maze (MWM)

The MWM is a test of spatial learning for rodents that relies on distal cues to navigate from start locations around the perimeter of an open swimming arena to locate a submerged escape platform. Spatial learning is assessed across repeated trials by the escape latency, and reference memory is determined by the preference for the former platform area when absent (probe trial). Mice were trained to find a 10 cm diameter platform submerged 1 cm below the surface of a pool measuring 120 cm in diameter and 50 cm in height, with non-reflective interior surfaces and filled with water made opaque using nontoxic white paint. The water temperature was maintained at 22 °C during all trials. The maze was surrounded by curtains and the same salient visual cues were used throughout the procedure. Mice were trained for 4 days with four trials per day at semi-randomly selected start positions. The interval between trials was approximately 20 min. The time from release until platform location was recorded as the escape latency for each trial. If the mice failed to find the platform within 60 s, they were guided to it and allowed to stay for ~15 s. A 60-s probe trial was performed 24 h after the final acquisition day without the escape platform, and the time spent in the former platform quadrant (target quadrant) was recorded as a measure of spatial memory.

### 2.4 Real-time quantitative polymerase chain reaction (RT-qPCR)

Total RNA was extracted from the tissue samples using the Tissue RNA Purification Kit PLUS (EZBioscience, USA) according to the manufacturer’s instructions and reverse transcribed into cDNA using HiScript II qRT SuperMix (Vazyme, China). Real-time quantitative PCR (RT-qPCR) was conducted using ChamQ™ SYBR® Colour PCR Master Mix (Vazyme, China) following the manufacturer’s instructions on a LightCycler 480 Instrument II. Relative expression levels of target genes were calculated based on the threshold cycle (Ct) using the 2^−ΔΔCt^ method and normalized to the expression of Gapdh. Primer sequences are listed in Supplementary Table S1.

### 2.5 Western blots

Tissues were lysed in ice cold RIPA lysis buffer (Beyotime, China) supplemented with a protease and phosphatase inhibitor cocktail, incubated on ice for 30 min, and centrifuged at 12,000 *g* for 15 min at 4 °C to obtain the supernatant. The supernatant protein concentration was measured using a Pierce™ BCA Protein Assay Kit (Thermo Fisher Scientific, USA). Equal amounts of protein were separated by sodium dodecyl sulphate-polyacrylamide gel electrophoresis (SDS-PAGE) and subsequently transferred to PVDF membranes. Membranes were blocked with 5% non-fat milk in Tris-buffered saline plus Tween 20 (TBST) buffer and incubated with the indicated primary antibodies overnight at 4 °C. Membranes were washed three times with TBST and then incubated with horseradish peroxidase (HRP)-conjugated anti-rabbit or anti-rat antibodies for 1 hour at room temperature. A detailed description of the antibodies can be found in Supplementary Table S2. The target proteins were detected by enhanced chemiluminescence, and expression levels were normalized by probing the same blots with *β*-actin or Gapdh antibody.

### 2.6 Enzyme-linked immunosorbent assay (ELISA)

Mouse whole blood samples were collected in EDTA-treated tubes and centrifuged at 2,000 rpm for 30 min at 4 °C to obtain plasma, while supernatants of tissue samples were prepared as described in Section 2.7. The concentrations of IL-1β, TNF-α, IL-6, and IFN-γ were measured in these samples using a Mouse ELISA Kit (Solarbio Sciences, China) according to the manufacturer’s instructions. In brief, diluted samples were added to kit microwells containing detection antibodies and incubated at room temperature for 2 hours on a microplate shaker. Microwells were then washed six times in wash buffer, with thorough aspiration of the contents between washes. Streptavidin-HRP was added to each well and allowed to react at room temperature for 45 min. After washing six times, TMB substrate solutions were added to each well and incubated for 30 min on a microplate shaker in darkness. The enzyme reaction was stopped by adding the stop solution, and optical density values were measured within 30 min on a spectrophotometer.

### 2.7 Immunofluorescence

Mice were deeply anesthetized with 2.0% isoflurane in a 30% oxygen/air mixture. Following loss of reflexes, the thoracic cavity was opened, and a perfusion cannula was inserted into the left ventricle. Transcardial perfusion was performed with 20 mL of ice-cold phosphate buffered saline (PBS), followed by 20 mL of freshly prepared 4% paraformaldehyde (PFA; Sigma-Aldrich) in PBS. Brains were removed, post fixed in 4% PFA at 4 °C overnight, and cryoprotected in 30% sucrose (w/v) in PBS at 4 °C until fully submerged. Tissues were embedded in optimal cutting temperature (OCT) compound (Sakura Finetek) and coronally sectioned at 25 μm using a cryostat (Leica CM1950). Sections were stored in antifreeze solution at −20 °C until use. For immunofluorescence, sections were washed in 0.3% PBST (PBS containing 0.3% Triton X-100), then permeabilized in 1% Triton X-100 in PBS for 30 min at room temperature (22 °C–25 °C). Non-specific binding was blocked using 5% normal serum (species matched to secondary antibody host; Jackson Immu-noResearch) and 0.1% Triton X-100 in PBS for 1 h at room temperature. Sections were incubated overnight at 4 °C with primary antibodies diluted in blocking buffer. After three PBS washes, sections were incubated with fluorophore conjugated secondary antibodies (Invitrogen) for 1.5–2 h at room temperature in the dark. After three final washes in PBS, sections were mounted with ProLong Glass Antifade Mountant (Thermo Fisher Scientific) and cover slipped. Details of antibodies are provided in Supplementary Table S2.

### 2.8 Label-free proteomics

#### 2.8.1 Protein extraction and trypsin digestion

Approximately 20–50 mg of wet-weight tissue per sample was lysed in TCEP buffer (2% deoxycholic acid sodium salt, 40 mM 2-chloroacetamide, 100 mM Tris–HCl, 10 mM Tris(2-chloroethyl) phosphate, 1 mM PFSM, 1 mM Cocktail, pH 8.5) supplemented with protease inhibitors and phosphatase at 99 °C for 30 min. After cooling to room temperature, trypsin (Promega, Madison, WI, USA) was added, and digestion was carried out for 18 h at 37 °C. Formic acid (10%) was added and vortexed for 3 min, followed by sedimentation for 5 min (12,000 g). Next, a new 1.5 mL tube containing extraction buffer (0.1% formic acid in 50% acetonitrile) was used to extract the supernatant (vortexed for 3 min, followed by sedimentation at 12,000 g for 5 min). The collected supernatant was then dried using a speedvac.

#### 2.8.2 SDS-page

A total of 20 μg of protein per sample was mixed with 2X loading buffer and boiled for 5 min. Proteins were separated on an SDS-PAGE gel (constant current 120 V, 60 min). Protein bands were visualized using Coomassie Blue R-250 staining.

#### 2.8.3 Nano-LC-MS/MS analysis

For proteome profiling, peptides were analyzed using the Orbitrap Ascend Hybrid Quadrupole-Orbitrap Mass Spectrometer (Thermo Fisher Scientific) coupled with a high-performance liquid chromatography system (Vanquish, Thermo Fisher Scientific). The Nano-LC-MS/MS analysis was performed by iProteome Co., Ltd. The iProteome one-stop data analysis cloud platform was used for qualitative and quantitative analysis. For the proteomic data, FOTs were multiplied by 1E5 for quantification, and missing values were imputed with 1E-5 and subsequently log₂ transformed, if necessary.

### 2.9 Standardized data curation and visualization analysis

All Data are presented as the mean ± standard error of the mean. Statistical significance was determined using an independent samples Student *t*-test, one-way ANOVA, or two-way ANOVA, as indicated, using GraphPad Prism 10 (San Diego, CA). A value of *p* < 0.05 was considered statistically significant for all tests.

Detailed methods are described in the Supplementary material.

## 3 Results

### 3.1 Low-dose intraperitoneal injection of LPS did not induce significant changes in inflammatory cytokine levels

**Figure 1:**
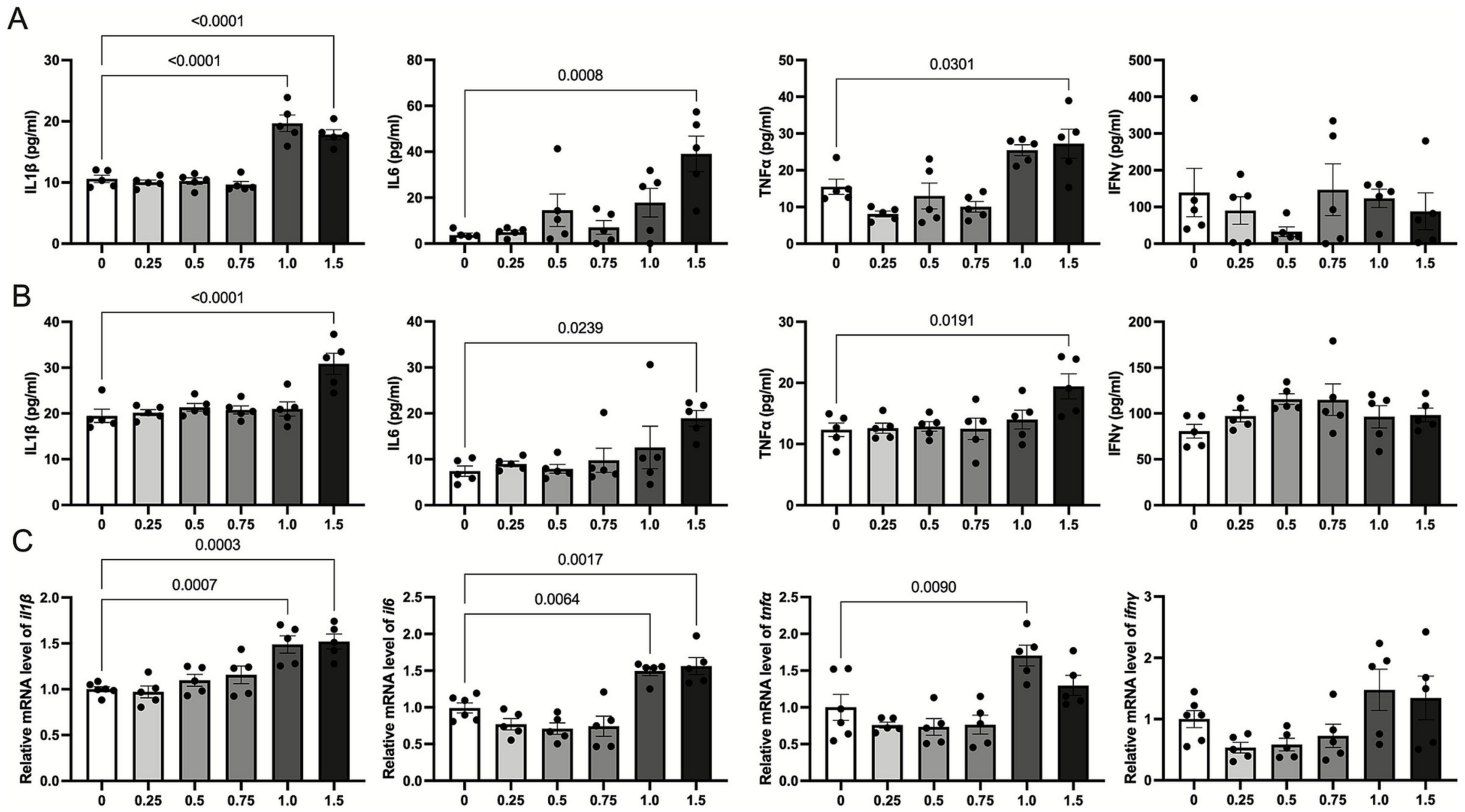
Effects of different doses of lps on inflammatory cytokine levels in plasma and hippocampal tissue. **(A)** Plasma levels of IL-1β, IL-6, TNF-α, and IFN-γ were measured 24 h after intraperitoneal injection of LPS at doses of 0, 0.25, 0.5, 0.75, 1, and 1.5 mg/kg using ELISA. High-dose LPS (1.5 mg/kg) significantly increased IL-1β, IL-6, and TNF-α levels, while IFN-γ levels remained unchanged. **(B)** ELISA analysis of hippocampal tissue revealed consistent results, showing significant increases in IL-1β, IL-6, and TNF-α levels with 1.5 mg/kg LPS, while IFN-γ levels showed no significant changes. **(C)** Relative mRNA expression levels of IL-1β, IL-6, TNF-α, and IFN-γ in hippocampal tissue were quantified by RT-qPCR. Doses above 1 mg/kg significantly upregulated IL-1β, IL-6, and TNF-α mRNA levels, while IFN-γ mRNA showed an upward trend without statistical significance. Data are presented as mean ± SEM, and statistical significance is indicated for relevant comparisons (*n* = 4–6 per group).

To determine the presurgical LPS dosage, we established six groups with different LPS concentrations (4–6 mice per group): 0, 0.25, 0.5, 0.75, 1, and 1.5 mg/kg. Twenty-four hours after LPS intraperitoneal injection, we measured the levels of inflammatory cytokines IL-1β, IL-6, TNF-α, and IFN-γ in plasma and hippocampus using ELISA and RT-qPCR, focusing on both protein and mRNA levels. The results revealed that intraperitoneal injection of a high LPS dose (1.5 mg/kg) significantly elevated plasma IL-1β, IL-6, and TNF-α levels after 24 h, while IFN-γ levels remained unchanged. At an LPS dose of 1 mg/kg, plasma IL-1β levels were significantly increased 24 h post injection. Conversely, LPS doses below 0.75 mg/kg did not induce significant changes in the levels of inflammatory cytokines in plasma after 24 h (Figure 1A). ELISA results from hippocampal tissue were consistent with the plasma findings; IL-1β, IL-6, and TNF-α levels were significantly increased 24 h after 1.5 mg/kg LPS administration, while IFN-γ levels showed no significant change (Figure 1B). We further assessed mRNA expression of IL-1β, IL-6, TNF-α, and IFN-γ in hippocampal tissue using RT-qPCR. The results showed that doses above 1 mg/kg significantly upregulated IL-1β, IL-6, TNF-α mRNA levels 24 h post injection, while IFN-γ mRNA expression showed an upward trend but without statistical significance (Figure 1C). Both ELISA and RT-qPCR results suggested that low dose LPS intraperitoneal injection triggers an inflammatory response in mice but does not lead to significant changes in cytokine levels. Based on these findings, we selected 0.75 mg/kg as the LPS concentration for priming the immune system in presurgical LPS infection experiments.

### 3.2 Low-dose intraperitoneal injection of LPS followed by anesthesia and surgery induced postoperative cognitive dysfunction

**Figure 2:**
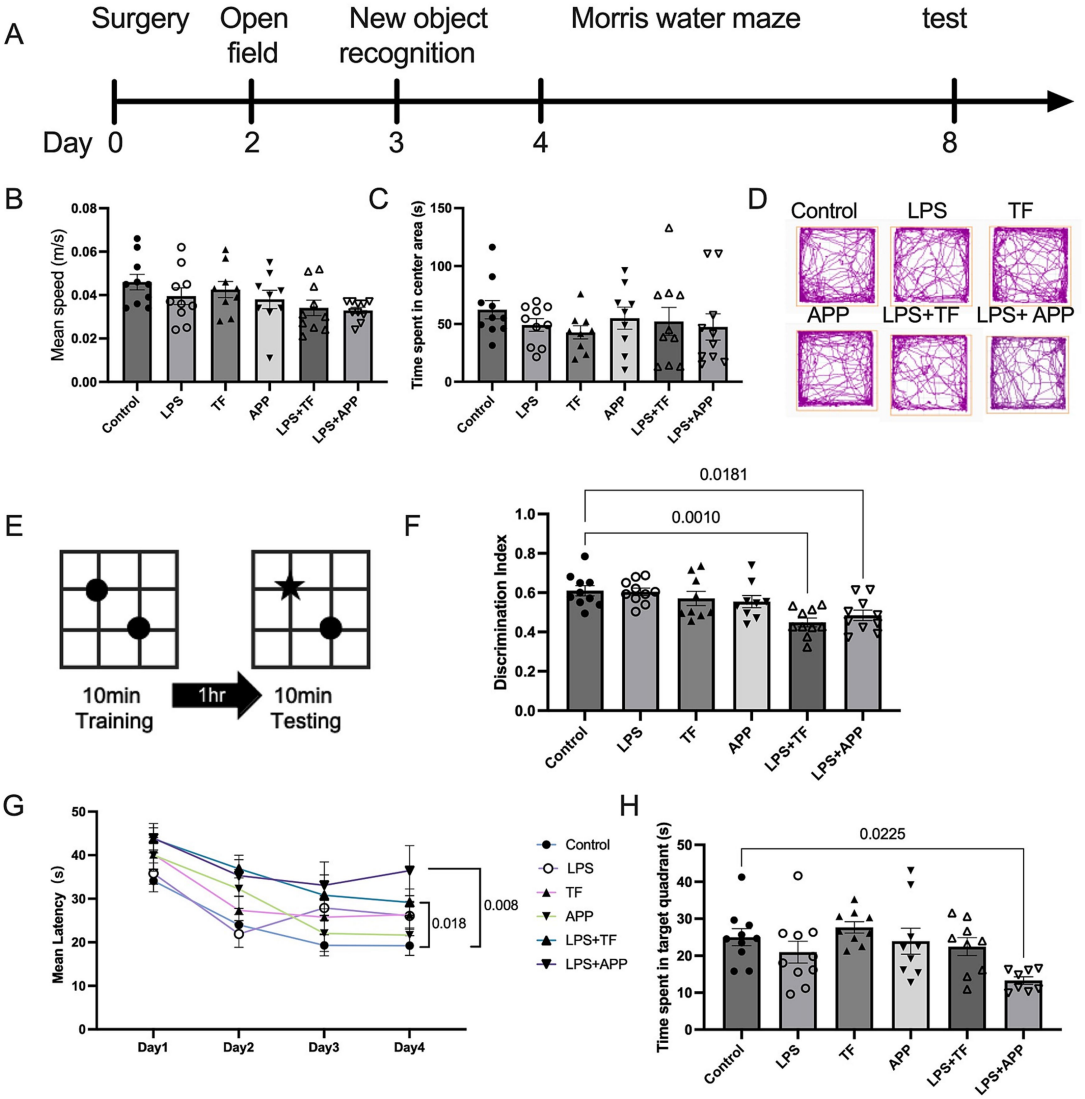
Low-dose LPS injection followed by surgery induces postoperative cognitive dysfunction in mice. **(A)** Experimental timeline illustrating the sequence of procedures and behavioral tests. **(B,C)** Open-field test (OFT) conducted on postoperative day 2. **(B)** Mean locomotor speed showed no significant differences among the six groups, indicating no impact on general activity levels. **(C)** Time spent in the centre zone was similar across groups, suggesting no evident anxiety-like behavior. **(D)** Representative traces of mouse movement during the OFT. **(E,F)** Novel Object Recognition (NOR) Test conducted on postoperative day 3. **(E)** Diagram of the training and testing phases. **(F)** Discrimination index of novel object exploration revealed significant deficits in the LPS + TF and LPS + APP groups compared with the control group. **(G,H)** Morris Water Maze (MWM) Test conducted on postoperative days 4–8 to assess spatial learning and memory. **(G)** Escape latency during the training phase. Mice in the LPS + TF and LPS + APP groups exhibited significantly longer escape latencies compared with other groups on the fourth training day. **(H)** Time spent in the target quadrant during the probe trial. The LPS + TF and LPS + APP groups spent significantly less time in the target quadrant compared with controls. Data are presented as mean ± SEM, with statistical significance indicated for relevant comparisons (*n* = 8–12 per group).

According to the literature, the peak effect of LPS occurs approximately 2 hours after intraperitoneal injection (Batista et al., 2019); therefore, the LPS + surgery groups received a 0.75 mg/kg i.p. injection of LPS 2 hours before undergoing surgery. In the LPS + surgery experiment, six groups of mice (*n* = 8–12/group) were established to investigate the effects of a subclinical dose of LPS administered prior to surgery. The control group received no treatment. Mice in the LPS group were administered an intraperitoneal injection of 0.75 mg/kg LPS. The TF group underwent tibial fracture fixation surgery, while the APP group underwent appendicectomy and partial caecum resection. Mice in the LPS + TF group received an intraperitoneal injection of 0.75 mg/kg LPS followed by tibial fracture fixation surgery. Similarly, mice in the LPS + APP group received an intraperitoneal injection of 0.75 mg/kg LPS followed by appendicectomy and partial caecum resection. The TF and APP groups were referred to as the surgery only group, while the LPS + TF and LPS + APP groups were collectively referred to as the LPS + surgery group in the subsequent sections. The detailed experimental timeline is presented in Figure 2A.

On the second postoperative day, an OFT was conducted. The results showed no significant differences in locomotor speed among the six groups, indicating that the experimental treatments did not affect the mice’s activity levels (Figure 2B). Additionally, the time spent in the central zone did not differ among the groups, suggesting no evident anxiety like or depressive behaviors following surgery (Figure 2C). Representative movement traces visually confirmed similar exploration patterns across all groups (Figure 2D).

On the third postoperative day, the NOR was conducted (Figure 2E). The results demonstrated that mice in the LPS + TF and LPS + APP groups spent significantly less time exploring the novel object compared with controls, with a markedly lower discrimination ratio (Figure 2F). In contrast, mice in the surgery only and low dose LPS groups performed comparably to controls. These findings suggested that postoperative memory impairment occurred in the LPS + TF and LPS + APP groups.

From the fourth postoperative day, the Morris Water Maze (MWM) test was conducted to assess spatial learning and memory. During the training phase, the latency to find the hidden platform progressively decreased in the control, LPS, TF, and APP groups. However, it remained persistently high in the LPS + TF and LPS + APP groups. By the fourth training day, escape latency in the LPS + TF and LPS + APP groups was significantly longer than in the other groups (Figure 2G). In the probe trial, mice in the LPS + TF and LPS + APP groups spent significantly less time in the target quadrant compared with the control group (Figure 2H). The results of the MWM and NOR tests indicated that neither low dose LPS nor surgery only (TF or APP) caused cognitive impairment in adult mice. However, low dose LPS followed by anesthesia and surgery led to postoperative cognitive dysfunction.

### 3.3 Low dose intraperitoneal injection of LPS followed by anesthesia and surgery resulted in a decrease in hippocampal synaptic protein

**Figure 3:**
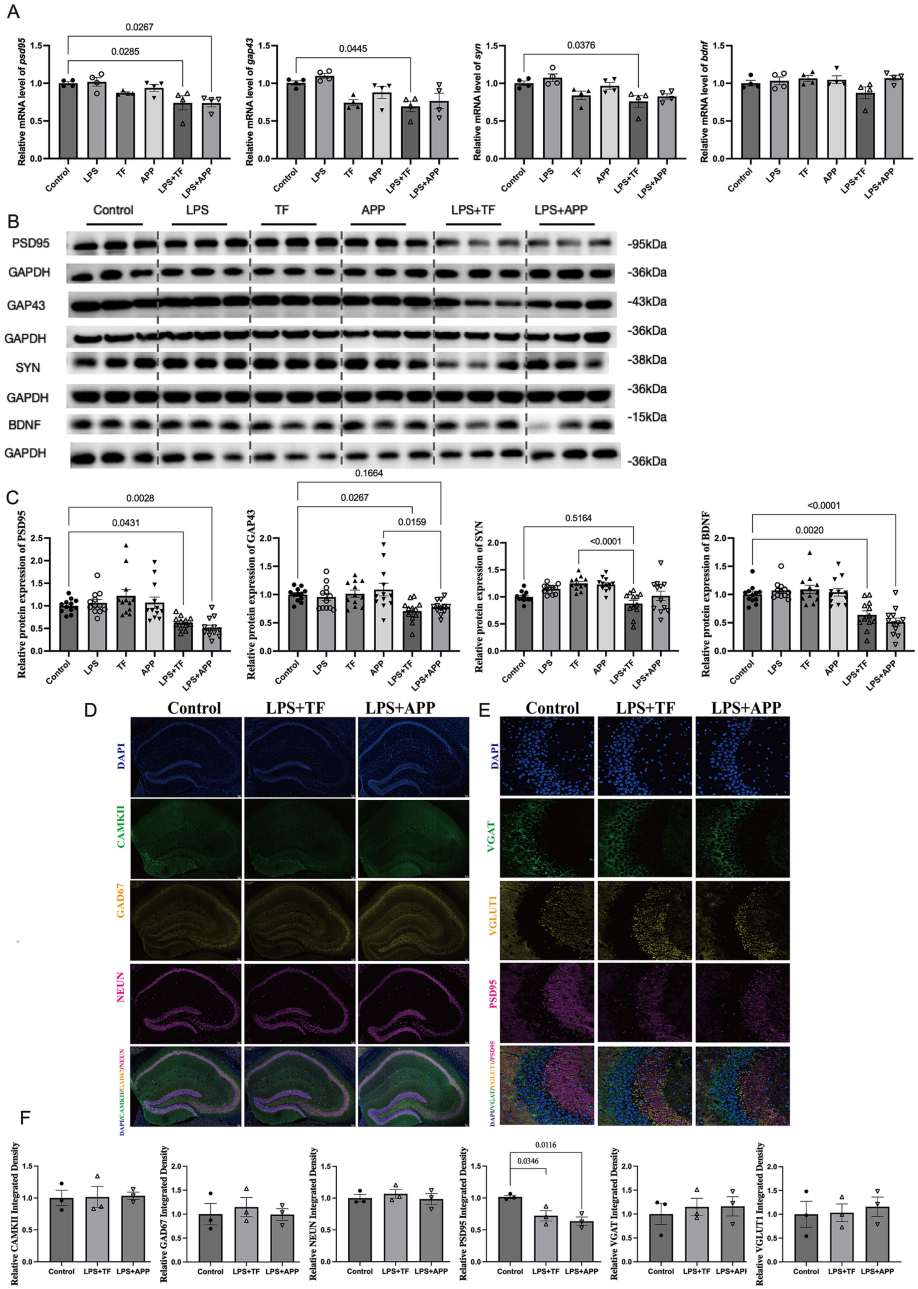
Low-dose LPS injection followed by surgery reduces hippocampal synaptic protein expression. **(A)** RT-qPCR of hippocampal Psd95, Gap43, Syn, and Bdnf mRNAs. Reductions in Psd95, Gap43, and Syn were observed in LPS + TF and LPS + APP; Bdnf mRNA was unchanged (exact contrasts annotated on plots). **(B)** Representative immunoblots for PSD95, GAP43, SYN, and mature BDNF. **(C)** Densitometry of the immunoblots (normalized to GAPDH) for the proteins in **(B)**. **(D)** Triple immunofluorescence in CA2: NeuN (total neurons), CaMKIIα (excitatory pyramidal neurons), and GAD67 (GABAergic interneurons). Cell-body densities were unchanged across groups. **(E)** Triple immunofluorescence in CA2 (synaptic markers): PSD95 (excitatory postsynaptic), VGLUT1 (excitatory presynaptic), and VGAT (inhibitory presynaptic). PSD95 puncta density was reduced in LPS + surgery group, whereas VGLUT1 and VGAT puncta were preserved. **(F)** Quantification for **(D,E)**: NeuN^+^, CaMKIIα^+^, GAD67^+^ cell densities and PSD95, VGLUT1, VGAT puncta densities. Data are presented as mean ± SEM, with statistical significance indicated for relevant comparisons (*n* = 3–12 per group).

To further investigate the effects of LPS + surgery on the central nervous system, we analyzed the expression of synaptic proteins (Postsynaptic Density Protein-95, PSD95; Growth Associated Protein 43, GAP43; Synaptophysin, SYN; and Brain Derived Neurotrophic Factor, BDNF) using RT-qPCR and Western blot. We found no significant changes in synaptic protein mRNA or protein levels in the low dose LPS group, or the surgery only groups (TF and APP) compared with the control group. However, in the LPS + surgery group, the mRNA levels of psd95, gap43, and syn were significantly reduced (Figure 3A), and the protein levels of PSD95 and mature BDNF were markedly decreased compared with the control group (Figures 3B,C). BDNF, which is approximately 30 kDa, exerts its functional effects when cleaved into mature BDNF. Although the mRNA level of bdnf remained unchanged, the level of mature BDNF (~15 kDa) was reduced (Rizzo et al., 2018). This finding suggests that preoperative low dose LPS treatment may impair the maturation of BDNF to some extent. For GAP43, densitometry showed a significant reduction in the LPS + APP group versus APP and LPS + TF group versus Control group, whereas the Control vs. LPS + APP comparison was not significant. For synaptophysin (SYN), levels were significantly lower in LPS + TF versus TF, while Control vs. LPS + TF was not significant. We found no evidence of neuronal loss: Densities of NeuN^+^, CaMKIIα^+^ (principal excitatory) and GAD67^+^ (inhibitory interneuron) cell bodies in hippocampus were unchanged across groups (Figures 3D,F). Selective postsynaptic change at excitatory synapses: PSD95 puncta density was reduced, whereas VGLUT1 (excitatory presynaptic) and VGAT (inhibitory presynaptic) puncta densities were not decreased (Figures 3E,F). These data argue against overt neurodegeneration of CA2 principal or inhibitory neurons and indicate a synaptic level alteration, specifically a postsynaptic weakening or destabilization of excitatory synapses (loss of PSD95 scaffolding) with preserved presynaptic terminal density (VGLUT1, VGAT unchanged).

### 3.4 Preoperative LPS treatment triggered inflammation the hippocampus

**Figure 4:**
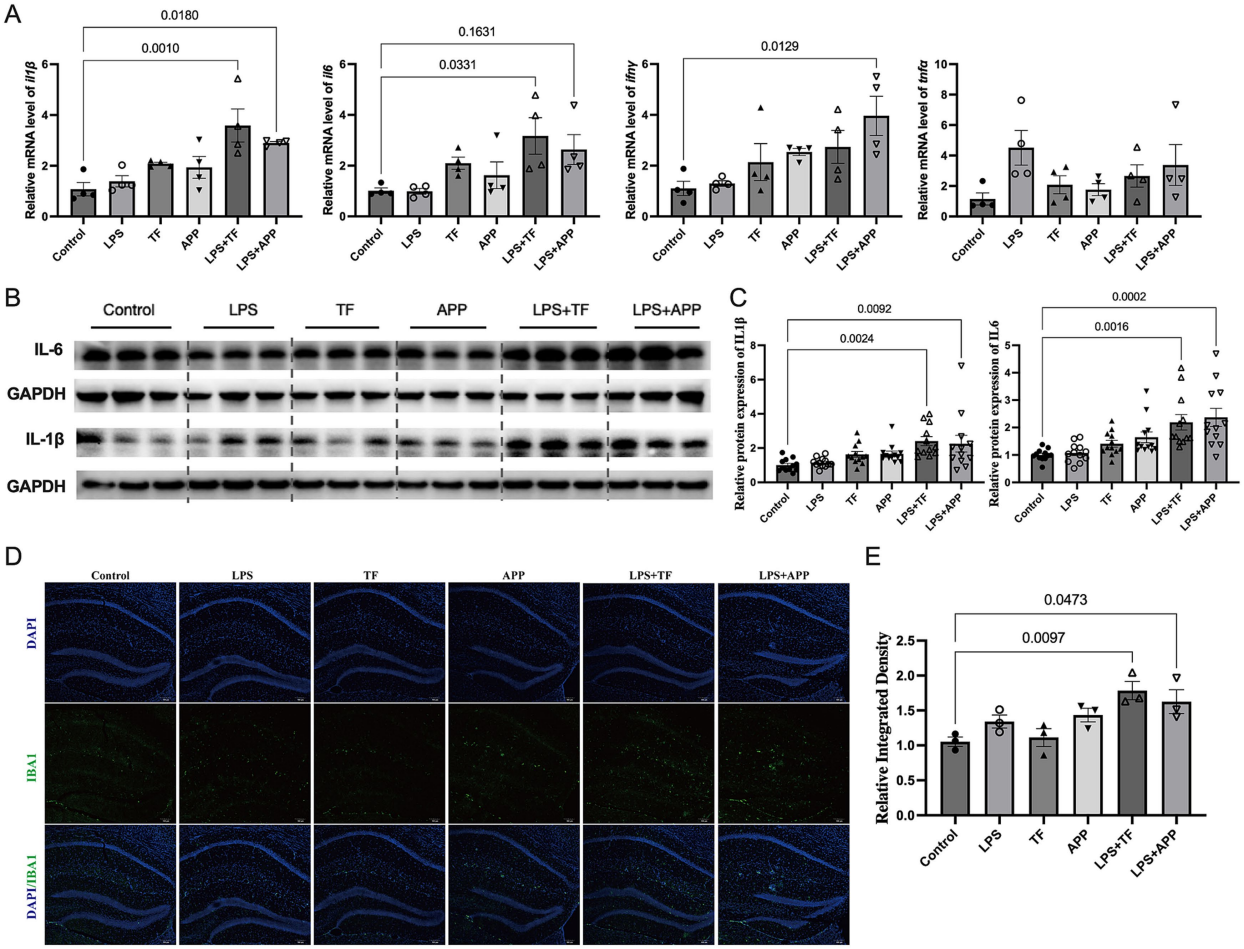
Preoperative LPS treatment primes the immune system and triggers hippocampal inflammation post-surgery. **(A)** Relative mRNA expression levels of inflammatory cytokines (IL-1β, IL-6, TNF-α, and IFN-γ) in hippocampal tissue were measured by RT-qPCR one day after surgery. Significant increases in IL-1β, IL-6, and TNF-α mRNA levels were observed in the LPS + TF and LPS + APP groups compared with controls, while IFN-γ levels remained unchanged. **(B)** Representative Western blot images of IL-1β and IL-6 protein levels in hippocampal tissue. GAPDH was used as a loading control. **(C)** Quantification of IL-1β and IL-6 protein levels relative to GAPDH. Significant elevations were observed in the LPS + TF and LPS + APP groups compared with the control group. **(D)** Representative immunofluorescence images of hippocampal tissue showing DAPI-stained nuclei (blue) and microglia marked by IBA1 (green) in each group. Merged images illustrate the distribution and activation of microglia. **(E)** Quantification of IBA1-positive microglial density in the hippocampus. The LPS + TF and LPS + APP groups exhibited significantly increased microglial activation compared with the control group. Data are presented as mean ± SEM, with statistical significance indicated for relevant comparisons (*n* = 3–12 per group).

To explore the effects of low dose LPS on the immune system and whether it could prime the immune system, we measured inflammatory cytokine levels (IL-1β, IL-6, TNF-α, and IFN-γ) in hippocampal tissue using RT-qPCR and Western blot 1 day after surgery in the control, LPS, TF, APP, LPS + TF, and LPS + APP groups. The results showed that 1 day post-surgery, cytokine levels in the hippocampus were not significantly altered in the low dose LPS, TF, or APP groups. In contrast, in the LPS + TF and LPS + APP groups, levels of IL-1β, IL-6, and TNF-α were significantly elevated in hippocampal tissue, while Ifng levels remained unchanged (Figures 4A–C). Immunofluorescence analysis of microglial activation in the hippocampus post-surgery revealed a marked increase in microglial cell numbers in the LPS + TF and LPS + APP groups (Figures 4D,E). These findings suggested that a subclinical dose of LPS could prime the immune system, and subsequent challenges, such as surgery, could amplify the pro-inflammatory response.

### 3.5 Potential mechanisms underlying cognitive impairment induced by LPS + APP

**Figure 5:**
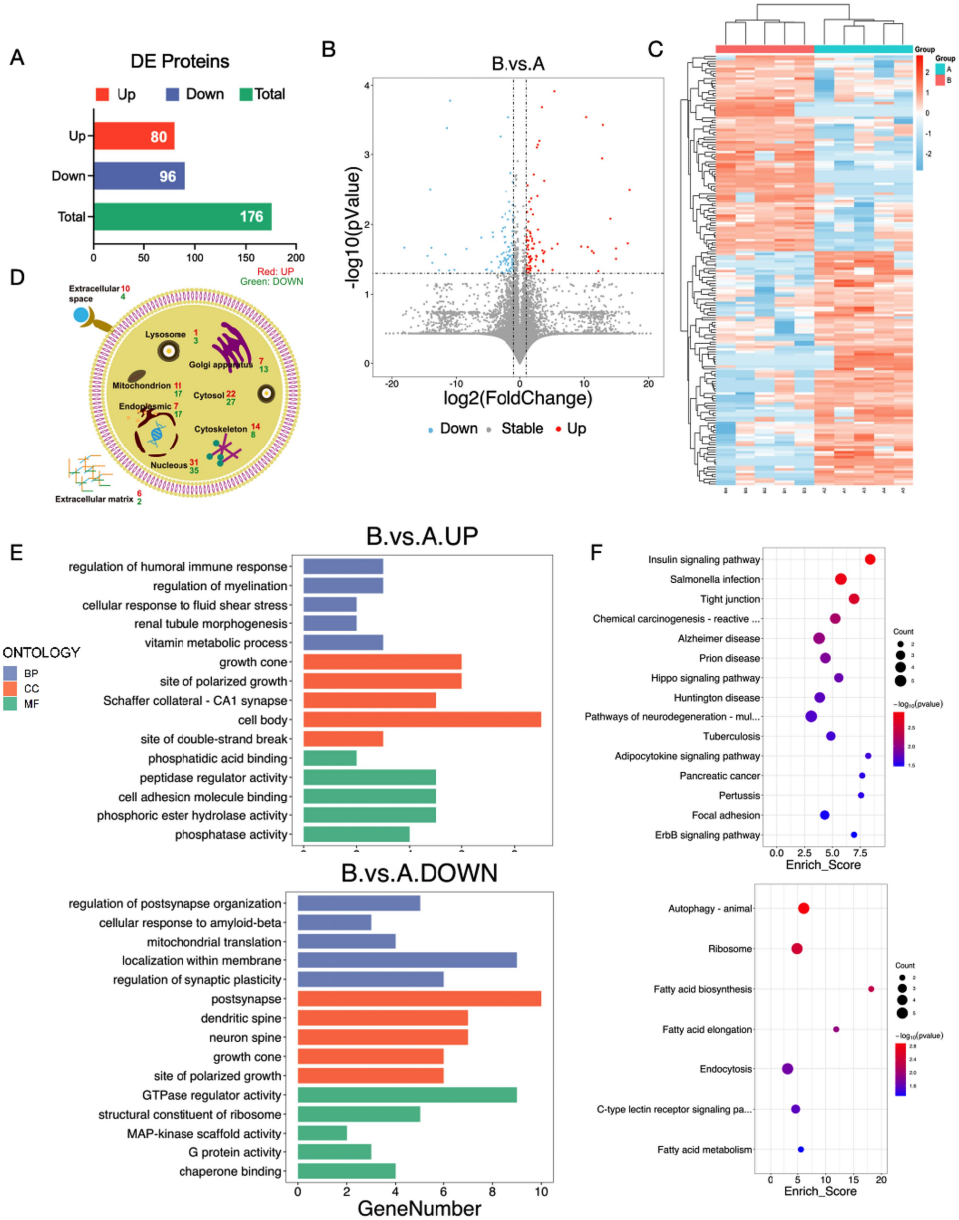
Label-free quantitative proteomics analysis reveals potential mechanisms underlying cognitive impairment induced by LPS + APP. **(A)** Bar graph showing the number of DEPs between the LPS + APP (surgery) group and the control group. A total of 176 DEPs were identified, including 80 upregulated and 96 downregulated proteins. **(B)** Volcano plot illustrating the distribution of DEPs, with significantly upregulated proteins marked in red and significantly downregulated proteins marked in blue. **(C)** Heatmap of hierarchical clustering analysis of DEPs. Distinct expression profiles were observed between the surgical and control groups. **(D)** Subcellular localization of DEPs, showing their distribution across organelles such as the nucleus, cytosol, mitochondria, endoplasmic reticulum, and extracellular matrix. Both upregulated and downregulated proteins were predominantly localized in the nucleus, cytosol, and mitochondria. **(E)** GO enrichment analysis of upregulated and downregulated proteins. **(F)** KEGG pathway enrichment analysis of DEPs. A: Control group; B: LPS + APP group.

To investigate the molecular mechanisms underlying the learning and memory impairment observed in the surgical mouse model, we conducted label-free quantitative proteomics on the hippocampal tissues of LPS + APP mice (hereafter referred to as the surgery group) and control mice. Using liquid chromatography–tandem mass spectrometry (LC-MS/MS), a total of 10,539 proteins were identified, among which 176 proteins were found to be differentially expressed between the surgical and control groups (*p* < 0.05), designated as differentially expressed proteins (DEPs). The final quantitative protein matrix is provided in the appendix. Of these, 80 proteins were significantly upregulated, and 96 proteins were significantly downregulated in the surgical group compared with the control group (Figure 5A). The clustering analysis revealed distinct expression profiles between the surgical and control groups, with the volcano and heatmap highlighting systematic differences in protein expression patterns (Figures 5B,C).

To investigate the subcellular localization of the DEPs, we performed subcellular annotation and quantified the distribution of these proteins across various organelles. The results showed that DEPs were predominantly localized in the nucleus (31 upregulated, 35 downregulated), cytosol (22 upregulated, 27 downregulated), mitochondria (11 upregulated, 17 downregulated), endoplasmic reticulum (17 upregulated, 17 downregulated), and cytoskeleton (14 upregulated, 8 downregulated). Additionally, differential expression was also observed in the extracellular matrix (6 upregulated, 2 downregulated) and extracellular space (10 upregulated, 4 downregulated) (Figure 5D). This distribution suggested that multiple organelles, particularly the nucleus, mitochondria, and endoplasmic reticulum, were significantly affected in the surgical group.

Gene Ontology (GO) enrichment analysis showed that the upregulated proteins in the surgery group were predominantly enriched in biological processes such as the regulation of humoral immune response, regulation of myelination, vitamin metabolic process, and growth cone related functions (Figure 5E). These findings suggested that surgical intervention might activate immune responses, promote synaptic remodeling, and enhance metabolic pathways, contributing to postoperative stress responses and repair processes. Conversely, the downregulated proteins were mainly associated with the regulation of postsynapse organization, mitochondrial translation, and GTPase regulator activity, indicating that surgery might suppress neuronal functional integrity and metabolic capacity, particularly impairing synaptic plasticity and energy metabolism. KEGG pathway analysis further elucidated the involvement of DEPs in critical signaling pathways (Figure 5F). Upregulated proteins were significantly enriched in pathways such as “Tight junction”, “Alzheimer’s disease”, and “Chemical carcinogenesis reactive oxygen species”, whereas downregulated proteins were enriched in pathways including “Autophagy – animal”, “Fatty acid metabolism”, and “Cytokine-cytokine receptor interaction”. These results suggested that surgical intervention may regulate hippocampal function and structure by activating metabolic pathways, tight junctions, and oxidative stress-related signaling, while simultaneously suppressing autophagy, lipid metabolism, and ribosomal function. This dual regulatory mechanism might serve as the molecular basis for the neurological changes and cognitive impairment observed after surgery.

**Figure 6:**
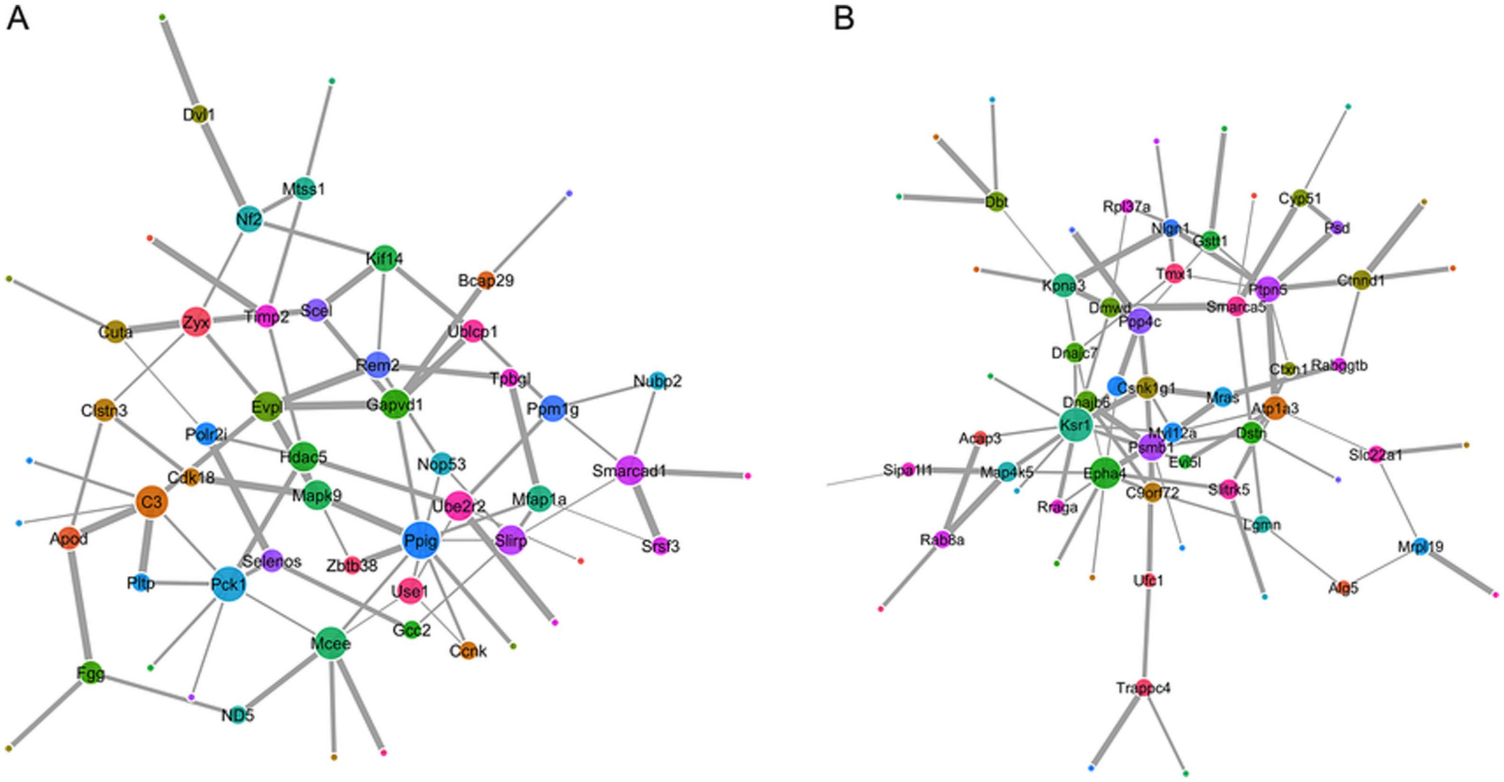
PPI network analysis of DEPs in the surgical model. **(A)** PPI network of upregulated proteins in the LPS + APP group compared with the control group. **(B)** PPI network of downregulated proteins in the LPS + APP group.

This protein-protein interaction (PPI) network illustrated the relationships among proteins affected in the surgical model. Each node represented a protein, with different colors indicating distinct functional categories or biological processes (e.g., signaling, metabolism, or inflammation), and node size reflecting its degree of interaction within the network. Edges between nodes indicated protein-protein interactions, with variations in thickness or length suggesting differences in interaction strength or confidence. PPI network analysis revealed key interactions among proteins upregulated (Figure 6A) or downregulated (Figure 6B) after surgery compared with the control group.

These findings provide molecular insights into the impact of surgery on hippocampal function and highlight potential targets for therapeutic intervention aimed at preserving cognitive integrity.

## 4 Discussion

The current study demonstrated that presurgical treatment with a subclinical dose of LPS, followed by surgical interventions, triggered peripheral and hippocampal inflammation, impaired synaptic protein expression, and led to cognitive dysfunction. This study also explored the conditions under which PND occurred in adult individuals and the impact of preoperative systemic inflammation on PND. A novel model of PND in adult mice was successfully established, providing a valuable tool for future research on PND.

Our findings indicated that the model established in this study shared key pathological features with classical PND models. Notably, both models exhibited heightened neuroinflammation and reductions in synaptic proteins, suggesting a common underlying mechanism contributing to postoperative cognitive impairment. LPS treatment following surgery significantly elevated the inflammatory response, as evidenced by increased IL-1β, IL-6, and TNF-α levels in plasma and hippocampal tissue in the LPS + surgery groups. These cytokines are known to mediate neuroinflammation, contributing to cognitive impairment by disrupting neuronal signaling and synaptic plasticity (Zhang et al., 2016; Rizzo et al., 2018; Bohlson and Tenner, 2023). Additionally, the increased microglial activation observed in the LPS + surgery group supports the hypothesis that LPS primes the central immune system, leading to exaggerated responses to subsequent challenges (Li et al., 2022). Neuroinflammation induced by anesthesia and surgery is a critical factor in the development of PND (Li et al., 2022). Following surgery, patients exhibit elevated levels of pro-inflammatory cytokines in both the systemic circulation and central nervous system, which correlate with the degree of cognitive decline (Qiao et al., 2015; Liu et al., 2023). The role of inflammation in the onset and progression of PND has gained increasing recognition.

This phenomenon mirrors findings in human studies, where pre-existing inflammation exacerbates PND, particularly in elderly or frail patients (Liu et al., 2023; Chen et al., 2020). This model also simulates real-world conditions in most emergency surgeries, as patients often undergo surgical procedures while already experiencing pre-existing inflammation due to various pathological conditions. These results underscore the potential role of preoperative inflammatory status as a critical determinant of surgical outcomes.

The reduction in PSD95, GAP43, and SYN protein levels in the hippocampus of LPS + surgery mice aligned with the observed deficits in spatial learning and memory in the MWM and NOR. PSD95 and GAP43 are integral to synaptic plasticity and memory formation, and their downregulation can directly impair synaptic function (Silva et al., 2024; Guang et al., 2018). Interestingly, while Bdnf mRNA levels remained unchanged, mature BDNF protein levels were significantly reduced in the LPS + surgery groups. As BDNF maturation is critical for synaptic plasticity and cognitive resilience, these findings suggest that LPS pretreatment disrupts post translational processes essential for BDNF functionality (Yang et al., 2020; Suri et al., 2013). The decline in mature BDNF could be linked to proteolytic imbalances or inflammatory mediators affecting its cleavage, which warrants further investigation. Consistent with the proteomics results, GO analysis of the proteomic data from the LPS + APP group post-surgery revealed a reduction in biological processes such as the regulation of post synapse organization and granulation of synaptic plasticity. There was also a decline in cellular components related to the post synapse, dendritic spine, and neuron spine, indicating impaired synaptic structure and function. In contrast, an increase was observed in the regulation of humoral immune response, reflecting heightened immune activity following the surgery. The sequencing results are provided in the attachment, which will aid in exploring the underlying mechanisms.

In the PPI network analysis, we identified several candidate molecules of interest that may guide future mechanistic investigations in this model. Among upregulated proteins, the most prominent hub proteins were MAPK9, DVL1, HDAC5 and APOD, all of which are closely associated with synaptic plasticity, neuronal signaling, and memory regulation. MAPK9, Mitogen-Activated Protein Kinase 9, is a critical component of the JNK signaling pathway, implicated in stress responses and synaptic modulation (Priori et al., 2023), both essential for cognitive processes. DVL1, Disheveled Segment Polarity Protein 1, functions as a key regulator of the Wnt signaling pathway (Rosso and Inestrosa, 2013), known to be involved in synaptic plasticity, neuronal survival, and cognitive function. HDAC5, Histone Deacetylase 5, is a nuclear cytoplasmic shuttling enzyme that regulates gene expression associated with learning and memory (Zhu et al., 2019). APOD, Apolipoprotein D, has been widely studied in the context of neuroprotection and cognitive disorders, potentially exerting its effects via lipid transport mechanisms (Dassati et al., 2014). Additionally, proteins such as TIMP2, C3, PCK1, and PLTP were also upregulated, suggesting their involvement in neuroinflammation, metabolic homeostasis, and synaptic regulation.

Among downregulated proteins, several hub proteins, including EPHA4, KPNA3, NLGN1, PTPN5, and SMARCA5, exhibited reduced expression, suggesting their involvement in synaptic integrity, neuronal signaling, and cognitive processing. EPHA4, Ephrin type-A receptor 4, a member of the Eph receptor family, plays a critical role in synaptic plasticity (Vargas et al., 2014) and axonal guidance, and its downregulation may contribute to deficits in synaptic connectivity (Richter et al., 2007). KPNA3, Karyopherin subunit alpha 3, is implicated in nuclear transport mechanisms crucial for neuronal function, and its reduction could disrupt transcriptional regulation of cognitive-related genes (Chen et al., 2020). NLGN1, Neuroligin 1, a key synaptic adhesion molecule, is essential for excitatory synapse formation and function, and its decreased expression suggests potential synaptic dysfunction (Scheiffele et al., 2000). Protein tyrosine phosphatase (PTP), non-receptor type 5, also known as STEP (STriatal-Enriched protein tyrosine Phosphatase), was the first brain-specific PTP discovered. PTPN5 is involved in dephosphorylation of key synaptic signaling molecules, and its reduction may alter synaptic signaling dynamics (Zhang et al., 2010). SMARCA5, a chromatin remodeling factor, is essential for neuronal differentiation and plasticity, and its downregulation may interfere with memory related gene expression (Qu et al., 2023). Additionally, proteins such as ATP1A3, MAP4K5, and CTXN1 were also found to be significantly downregulated, indicating possible disruptions in ion homeostasis, cytoskeletal dynamics, and intracellular trafficking, all of which are essential for synaptic stability and cognitive function.

Wang et al. (2021) performed upper mesenteric artery clamping surgery (UMAC) on 12-week-old adult Wistar rats to simulate adult PND. Compared with this model, our LPS-primed surgery model offers greater specificity in studying PND. The UMAC model induces systemic inflammation but also involves intestinal mucosal barrier damage and gut microbiota translocation, introducing confounding factors. In contrast, our model isolates the effects of preoperative immune priming, allowing a clearer assessment of the relationship between systemic inflammation, neuroinflammation, and synaptic dysfunction. It is also more practical and reproducible than the UMAC model, as it avoids complex surgical procedures. Fidalgo et al. (2011) investigated the impact of subclinical infection on surgery-induced cognitive decline. In their study, mice first underwent fear-conditioning training, followed by a 50 ng/kg intraperitoneal (i.p.) injection of LPS administered 2 hours before tibia surgery, 1 day after training. Postoperative fear memory was assessed on the following day. Their findings are consistent with ours, suggesting that subclinical infection may sensitize the immune system, thereby exacerbating the severity of postoperative cognitive dysfunction. However, in their experiment, preoperative fear-conditioning training introduced significant stress to the mice. This means their study assessed the combined effects of stress, subclinical infection, and surgery on cognitive function, rather than isolating the impact of subclinical infection and surgery alone. Moreover, their study focused solely on the assessment of fear memory and did not explore the potential underlying mechanisms contributing to the observed cognitive dysfunction.

While this study provided valuable insights into the impact of preoperative subclinical infection on PND, there were certain limitations that should be acknowledged. We did not further investigate the detailed pathophysiological mechanisms underlying PND in adult mice. Although our findings suggested that neuroinflammation and synaptic dysfunction play a role, additional studies are needed to elucidate the precise molecular pathways involved. Our model utilized LPS as the sole inflammatory agent to prime the immune system. While LPS is widely used to mimic systemic infection, other pro-inflammatory stimuli, such as cytokine infusion or viral mimetics, could provide a broader understanding of how different inflammatory triggers contribute to PND. Additionally, in clinical settings, PND is influenced by multiple factors beyond inflammation, including sleep deprivation, psychological stress, and metabolic disturbances. Our study did not explore the potential interactions between these factors and surgery induced neurocognitive impairment, which could be relevant for a more comprehensive understanding of PND pathogenesis.

## 5 Conclusion

In conclusion, the current study successfully established a novel model of PND in adult mice. The study demonstrated that administering a low dose LPS followed by surgery induced an inflammatory response, leading to postoperative impairments in learning and memory. Our findings suggested that subclinical infection may sensitize the immune system, thereby augmenting the severity of postoperative cognitive dysfunction.

## Data Availability

The datasets presented in this study can be found in online repositories. The names of the repository/repositories and accession number(s) can be found in the article/Supplementary material.
