# Supplementary material for: Subclinical infection combined with surgery induced cognitive dysfunction: a novel adult mouse model for perioperative neurocognitive disorder
Source: Front Aging Neurosci. 2026 Jan 12;17:1691681. doi: 10.3389/fnagi.2025.1691681 (PMC12832885; doi:10.3389/fnagi.2025.1691681)
Supplement: Supplementary file 1 [file Supplementary_file_1.pdf]

Supporting information

Supplementary Table 1 Primer sequences for RT-qPCR

Gene	Forward Primer Sequence (5'-3')	Reverse Primer Sequence (5'-3')
Gapdh	AAGAAGGTGGTGAAGCAGGCATC	CGGCATCGAAGGTGGAAGAGTG
Ifng	ATGAACGCTACACACTGCATC	CCATCCTTTTGCCAGTTCCTC
Tnf	CAGGCGGTGCCTATGTCTC	CGATCACCCCGAAGTTCAGTAG
il6	TAGTCCTTCCTACCCCAATTTCC	TTGGTCCTTAGCCACTCCTTC
il1b	GAAATGCCACCTTTTGACAGTG	TGGATGCTCTCATCAGGACAG
Psd95	GGTCAGCCCTCTGGCTACT	GTCCGTGTTGACAATCACAGG
Syn1	CAGTTCCGGGTGGTCAAGG	ACTCTCCGTCTTGTTGGCAC
Bdnf	TCATACTTCGGTTGCATGAAGG	AGACCTCTCGAACCTGCCC
Gap43	TGGTGTCAAGCCGGAAGATAA	GCTGGTGCATCACCCTTCT

Supplementary Table 2. Detailed information regarding antibodies and reagents.

Antibody/Reagent Name	Catalog No	Brand
Anti-GAP43 Antibody	ab75810	Abcam
BDNF Polyclonal Antibody	PA5-111802	Thermo Fisher
Anti-Synaptophysin Antibody	ab32127	Abcam
PSD-95 Polyclonal Antibody	516900	Thermo Fisher
Anti-PSD95 antibody	ab238135	Abcam
Gad67 Guinea pig pAb	OB-PGP074-02	Oasis Biofarm
Anti-SLC32A1/VGAT antibody	ab307448	Abcam
Anti-NeuN antibody	ab177487	Abcam
Anti-CaMKII-α mouse mAb	50049	CST
SLC17A7/vGluT1 Guinea pig pAb	OB-PGP101-01	Oasis Biofarm
Anti-IL-6 Rabbit pAb	GB11117-100	Servicebio
Anti-IL-1 beta Mouse mAb	A22257	Abclonal
HRP Anti-beta Actin	Ab49900	Abcam
HRP Anti-GAPDH	ZB15004-HRP-100	Servicebio
HRP Anti-GAPDH	ZB15004-HRP-100	Servicebio
Anti-Iba1 Guinea pig pAb	OB-PGP049	Oasis Biofarm
Goat-anti-Guinea pig IgG, AF488	G-GP488	Oasis Biofarm
Donkey-anti-Guinea pig IgG, AF555	D-GP555	Oasis Biofarm
Donkey-anti-Rabbit IgG, AF647	D-RB647	Oasis Biofarm
Donkey-anti-Mouse IgG, AF488	D-MS488	Oasis Biofarm
Lipopolysaccharide Escherichia coli (O111:B4)	L2630	Sigma-Aldrich
