# Supplementary figures and images for: Subclinical infection combined with surgery induced cognitive dysfunction: a novel adult mouse model for perioperative neurocognitive disorder
Source: Front Aging Neurosci. 2026 Jan 12;17:1691681. doi: 10.3389/fnagi.2025.1691681 (PMC12832885; doi:10.3389/fnagi.2025.1691681)

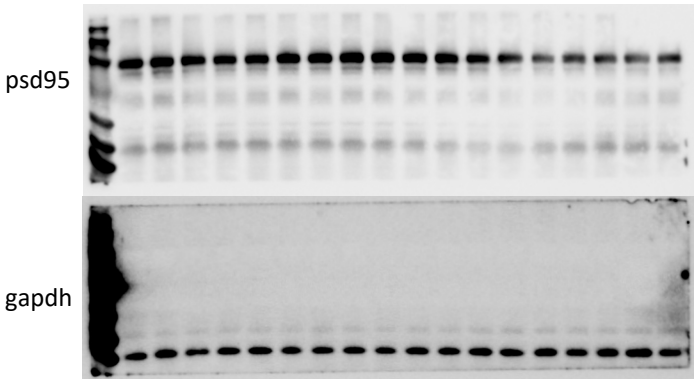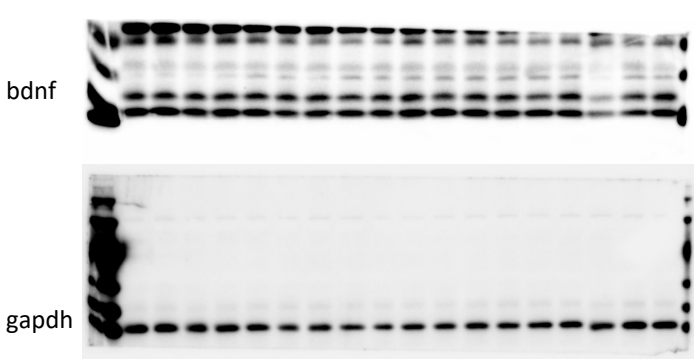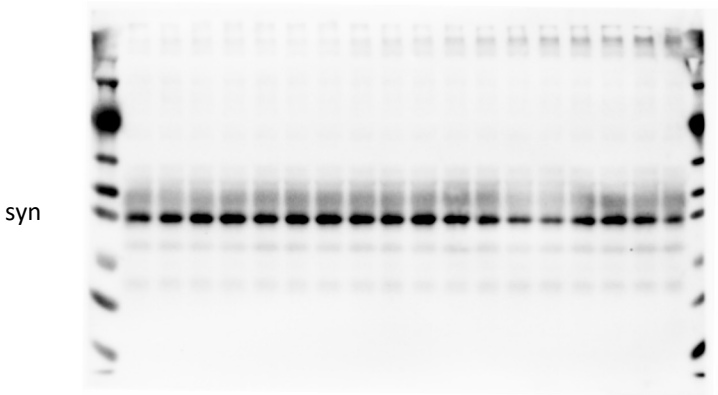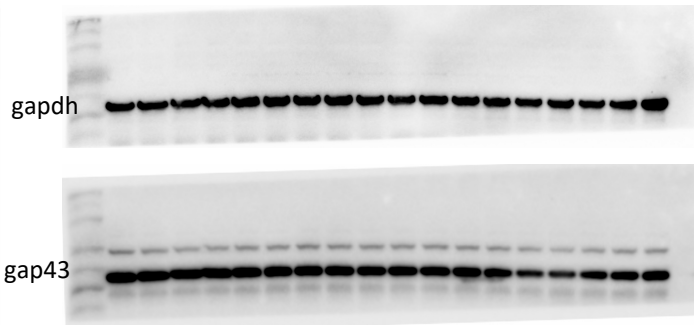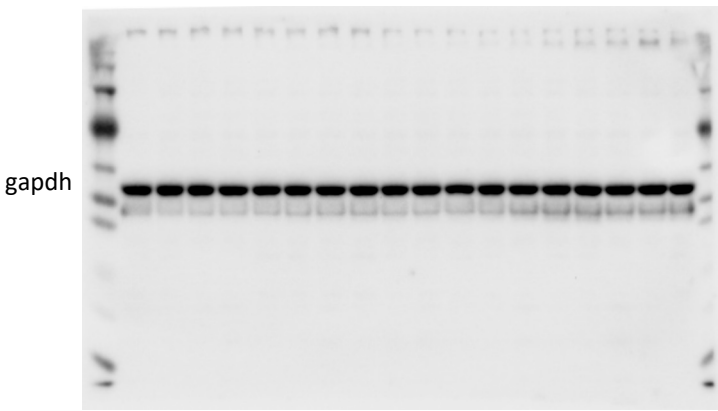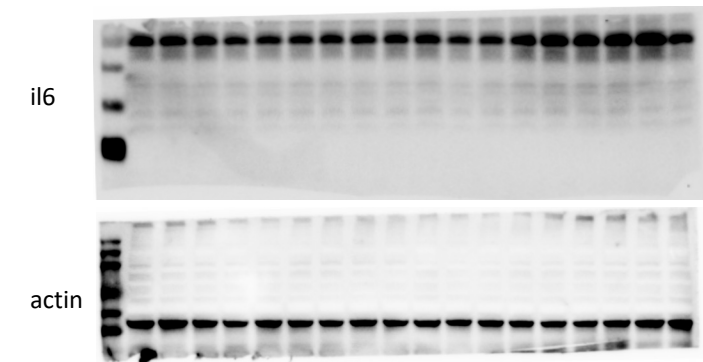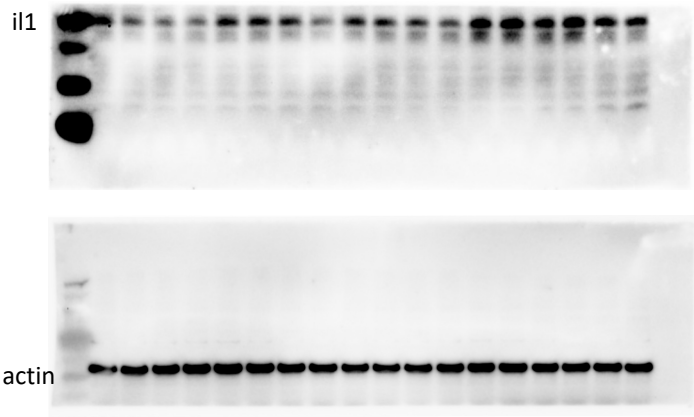

Supplement: Supplementary file 2 [file Supplementary_file_2.pdf]
